# Non-Enzymatic Electrochemical Sensor Based on Sliver Nanoparticle-Decorated Carbon Nanotubes

**DOI:** 10.3390/molecules24183411

**Published:** 2019-09-19

**Authors:** Dongqing Xu, Bingbing Hou, Lisheng Qian, Xueji Zhang, Guodong Liu

**Affiliations:** 1 Institute of Biomedical and Health Science, School of Life and Health Science, Anhui Science and Technology University, Fengyang 233100, Anhui, China; xudq@ahstu.edu.cn (D.X.); Houbb@ahstu.edu.cn (B.H.); 2 School of Biomedical Engineering, Shenzhen University Healthy Science Center, Shenzhen 518060, Guangdong, China

**Keywords:** carbon nanotubes, silver nanoparticles, nonenzymatic, sensor, hydrogen peroxide

## Abstract

The authors report a non-enzymatic electrochemical sensor based on a sliver nanoparticle-decorated carbon nanotube (AgNPs-MWCNT). Highly-dispersed AgNPs were loaded on the MWCNT surface though a simple and facile two-step method. The morphology, components, and the size of the AgNPs-MWCNT nanocomposite were characterized by transmission electron microscopy, X-ray diffraction, and ICP analysis. Benefitting from the synergistic effect between the AgNPs and MWCNT, the AgNPs-MWCNT nanocomposite exhibited high electrocatalytic activity for H_2_O_2_; the AgNPs-MWCNT electrochemical sensor was prepared by coating the AgNPs-MWCNT nanocomposite on a glassy carbon electrode, and it showed a fast and sensitive response to H_2_O_2_ with a linear range of 1 to 1000 μM. The detection limit was 0.38 μM (S/N = 3). The sensor was applied to detect H_2_O_2_ in spiked human blood serum samples with satisfactory results.

## 1. Introduction

Carbon nanotubes (CNTs) as perfect one-dimension nanomaterials are widely applied to various fields including energy storage, heterogeneous catalysis, sensors, biosensors, and DNA theranostic, due to their excellent physicochemical performance, unique electrochemical properties, and easy functionalization [1,2,3,4,5,6]. CNTs are easily functionalized through physical ultraphonic, chemical acidification and oxidation. The functionalized multi-wall carbon nanotubes (MWCNTs) have been used as carriers to immobilize various nanoparticles and biological molecules for various applications, such as biosensors and bioassays [7,8]. Silver is a low-cost noble metal and silver nanostructures have remarkable catalytic activity and good electron transfer efficiency [9,10,11]. Silver nanostructures, including nanoparticles and nanotubes, are used to construct electrochemical sensors for the ultrasensitive detection of hydrogen peroxide [12,13], proteins [14], lactose [15], glucose [16], and hydroquinone [17]. Due to the unique properties of CNT and silver nanoparticles, the preparation of CNT/AgNPs hybrid nanocomposites has received considerable interest [18,19,20]. Benefitting from the synergistic effect between the AgNPs and CNT, the AgNPs/CNT hybrid nanocomposite exhibits excellent performance on electron transfer and electrocatalytic activity. Various approaches including sputter deposition [21], silver mirror reaction [22], template self-volatilization [23], one-step hydrothermal method [24], electrostatic adsorption [25,26], and in-situ chemical reduction [27], were used to prepare the AgNPs-loaded CNT (AgNPs-CNT) nanocomposites. The AgNPs-CNT nanocomposite, prepared with the electrostatic adsorption method, has less AgNPs on the CNT surface, which influences its catalytic activity. The in-situ chemical reduction of Ag^+^ ions in dispersed CNT solution would increase the amount of AgNPs on the CNT surface, but the AgNPs possess irregular morphology and size due to the uncontrolled nucleation and growth processes. Moreover, CNT aggregates in aqueous solutions, which affects its performance and applications. Although, many methods and techniques are used to prepare AgNPs-CNT nanocomposites, there is still a challenge to develop a simple and mature method for the controllable growth of AgNPs on carbon nanotubes substrate.

In this article, the highly-dispersed AgNPs with a diameter of 12 nm were loaded on MWCNTs though a simple and facile two-step procedure. The morphology and size of the AgNPs-MWCNT nanocomposite were characterized by transmission electron microscopy (TEM), X-ray diffraction, and ICP analysis. The AgNPs-MWCNT nanocomposite was used to develop a nonenzymatic electrochemical sensor for the detection of hydrogen peroxide (H_2_O_2_). H_2_O_2_ is widely used as an oxidizing agent in many fields, such as the clinical, pharmaceutical, and food industries. In addition, H_2_O_2_ is an important marker for oxidative stress and the side product of oxidase enzyme metabolism. Hence, the determination of H_2_O_2_ in trace levels in biological and various water samples are of great importance. Electrocatalytic activity of AgNPs-MWCNT nanocomposite to H_2_O_2_ was studied by cyclic voltammetry and amperometry was used to quantify the concentration of H_2_O_2_. The sensor was applied to detect H_2_O_2_ in spiked serum samples.

## 2. Results and Discussion

### 2.1. Characterization of AgNPs-MWCNT Nanocomposite

**Figure 1:**
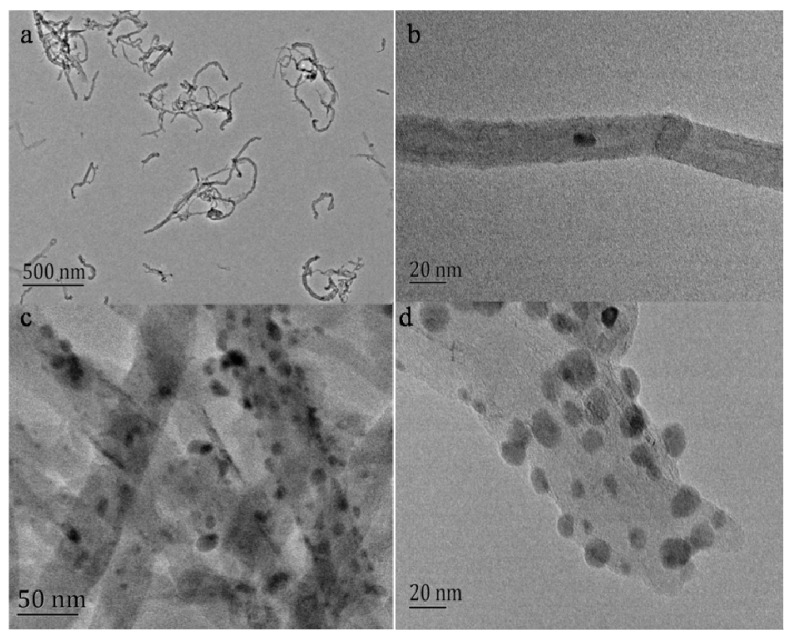
Typical TEM images of the shortened MWCNTs (**a** and **b**) and the synthesized AgNPs-MWCNT nanocomposites (**c** and **d**).

The AgNPs-MWCNT nanocomposite was synthesized through a two-step route. First, MWCNTs were treated with strong acids to shorten the length and introduce carboxylic groups on the CNT surface. Second, the mono-dispersed AgNPs were decorated on the MWCNTs’ surface by the in-situ reduction of AgNO_3_ with sodium citrate. The size and morphology of the as-prepared AgNPs-MWCNT nanocomposite were characterized by transmission electron microscopy (TEM). Figure 1a,b are the TEM images of the shortened MWCNTs. One can see the length of the MWCNTs is about 200 nm (Figure 1a) and the diameter of the MWCNTs is around 20 nm (Figure 1b). Figure 1c presents the typical TEM image of the AgNPs-MWCNT nanocomposites. Large amounts of AgNPs were loaded on the MWCNTs’ surface and the distribution of AgNPs on the MWCNTs’ surface is uniform. The diameter of AgNPs ranges from 8 to 13 nm (Figure 1d). The components of the AgNPs-MWCNT were determined by inductively coupled plasma atomic emission spectrometer (ICP-AES) and the Ag content in the as-prepared AgNPs-MWCNT nanocomposite is 0.2356 wt%.

**Figure 2:**
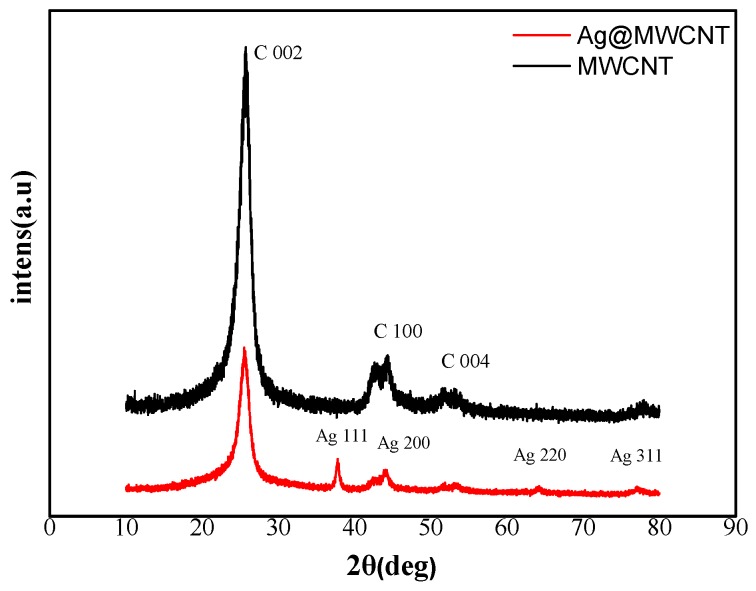
XRD patterns of the shortened MWCNT (black) and AgNPs-MWCNT nanocomposite (red).

X-ray diffraction (XRD) was used to study the crystalline phases of the MWCNTs and AgNPs-MWCNT nanocomposite. As shown in Figure 2, the diffraction peaks of MWCNTs (black) at 25.8°, 43.5°, and 52.5° in the XRD pattern correspond to the lattice spacing of the carbon atom (002), (100), and (004), respectively [28]. The diffraction peaks of the AgNPs-MWCNT nanocomposite (red) at 38°, 44°, 64°, and 78° in XRD pattern ascribes to Ag (111), (200), (220), and (311), respectively. The above XRD pattern proves that the AgNPs-MWCNT nanocomposite was successfully synthesized [29].

### 2.2. Electrocatalytic Activity of AgNPs-MWCNT Nanocomposite to H_2_O_2_

**Figure 3:**
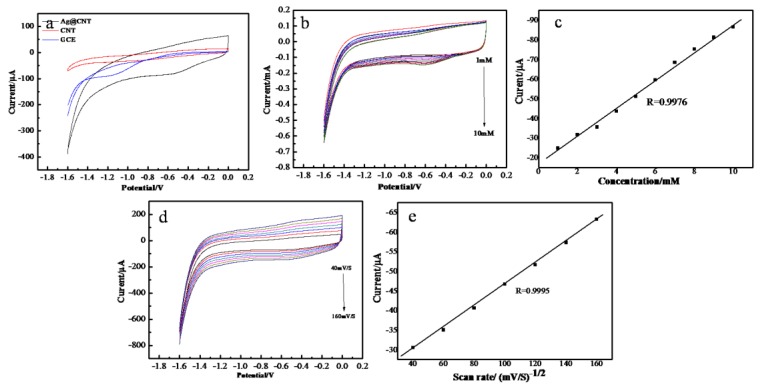
(**a**) Cyclic voltammograms of GCE, MWCNT/GCE, and AgNPs-CNT/GCE in N_2_-saturated 0.1M PBS solution containing 5 mM H_2_O_2_, potential scan rate: 100 mV/s; (**b**) cyclic voltammograms of AgNPs-MWCNT/GCE in N_2_-saturated 0.1M PBS in the presence of H_2_O_2_ with different concentrations (1, 2, 3, 4, 5, 6, 7, 8, 9, and 10 mM), potential scan rate: 100 mV/s; (**c**) relationship between the reduction peak current and H_2_O_2_ concentrations. (**d**) Cyclic voltammograms of AgNPs-MWCNT/GCE in N_2_-saturated 0.1 M PBS containing 1 mM H_2_O_2_ at different scan rates (40, 60, 80, 100, 120, 140, and 160 mV/s); (**e**) relationship between the peak current vs. square root of potential scan rate.

The AgNPs-MWCNT nanocomposite was used to prepare a nonenzymatic electrochemical sensor for the detection of H_2_O_2_. The electrocatalytic activity of the AgNPs-MWCNT nanocomposite to H_2_O_2_ was studied by cyclic voltammetry (CV). Figure 3a shows the typical cyclic voltammograms of the bare glassy carbon electrode (GCE), MWCNT modified GCE (MWCNT/GCE), and AgNPs-MWCNT modified GCE (AgNPs-MWCNT/GCE) in 0.1 M phosphate buffer saline (PBS, pH 7.4) containing 5 mM H_2_O_2_ at a potential scan rate of 100 mV/s. As shown in Figure 3a, there is no redox peak observed on the bare GCE electrode; an obvious reduction peak (peak potential: −0.55 V) was found with AgNPs-MWCNT/GCE, and the peak current was around −81.3 μA, which is much higher than that obtained with MWCNT/GCE. The above results show that the H_2_O_2_ were reduced at the AgNPs-MWCNT/GCE surface effectively, and the AgNPs-MWCNT has excellent electrocatalytic activity to the reduction of H_2_O_2_. The electrocatalytic mechanism of AgNPs-MWCNT to H_2_O_2_ reduction can be illustrated as follows [30,31]:

$$H_{2}O_{2}+2e\;\overset{\mathrm{AgNP}(\mathrm{pH}\;7.4)}{\to }{\;2\mathrm{OH}}^{-}$$

$${2\mathrm{OH}}^{-}\;\overset{}{\to }{\;H}_{2}O+\frac{1}{2}O_{2}+2e$$

The AgNPs on the MWCNT’s surface accelerate the reduction of H_2_O_2_ to generate hydroxide ions (OH^−^). The reduction peak potential of H_2_O_2_ shifts from −0.85 V on MWCNT/GCE to −0.55 V on AgNPs-MWCNT/GCE. The enhanced reduction current and the shifts of peak potential indicate that the AgNPs-MWCNT nanocomposite has excellent electrocatalytic activities to H_2_O_2_ [24]. Figure 3b presents the CV curves of the AgNPs-MWCNT/GCE in the presence of different concentrations of H_2_O_2_ (0 to 10 mM). One can see the peak current at −0.55 V increases with the increase of H_2_O_2_ concentrations. There is a linear relationship (R = 0.9976) between the H_2_O_2_ concentration and the peak current (Figure 3c), indicating that the AgNPs-MWCNT/GCE can be used as a nonenzyme electrochemical sensor for the detection of H_2_O_2_. The effect of the potential scan rate on the peak current was studied by varying the potential scan rate from 40 to 160 mV/s. Figure 3d presents the cyclic voltammograms of the AgNPs-MWCNT/GCE under the different potential scan rates. The reduction peak currents increase with the increase of potential scan rates. There is a good linear relationship between the square root of potential scan rate and the peak current (Figure 3e) and the correlation coefficient is 0.9989, indicating a classic diffusion-controlled process of H_2_O_2_ on AgNPs-MWCNT/GCE [32].

### 2.3. Amperommetric Response of H_2_O_2_ on AgNPs-MWCNT/GCE

**Figure 4:**
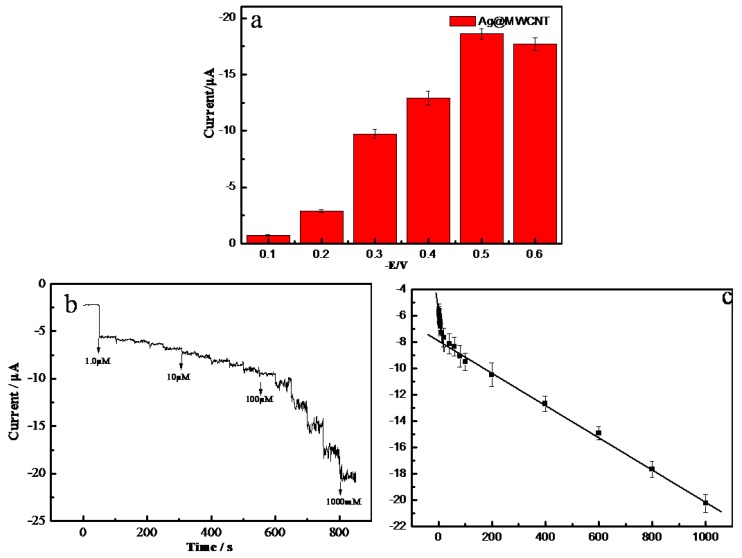
(**a**) The responsive currents of AgNPs-MWCNT/GCE in the presence of 1 mM H_2_O_2_ at different working potential from −0.1 to −0.6 V with 0.1 V interval; (**b**) typical amperommetric responses of AgNPs-MWCNT/GCE to the successive addition of H_2_O_2_ in 0.1 M PBS at working potential of −0.5 V; (**c**) the dependence of the responses of electrodes on H_2_O_2_ concentrations.

Amperommetry was used to inspect the possibility of the AgNPs-MWCNT/GCE as a nonenzymatic electrochemical sensor for the detection of H_2_O_2_. In order to obtain the optimum working potential, the amperommetric current of 1.0 mM H_2_O_2_ on AgNPs-MWCNT/GCE was measured at the working potential ranging from −0.1 to −0.6 V with an interval of 0.1 V (Figure 4a). It can be seen that the amperommetric response of H_2_O_2_ increased with the decrease of the potentials, and the highest response was obtained at −0.5 V. A further decrease of the working potential led to a decrease of the current. Therefore, a potential of −0.5 V was selected as the optimum potential for the amperommetric detection of H_2_O_2_. We aimed to detect H_2_O_2_ in the blood or the physiological system with the AgNPs-MWCNT/GCE sensor, so we chose the pH value of 7.4 to match the physiological condition without further optimization. The AgNPs-MWCNT with the concentrations ranging from 1 mg mL^−1^ to 5 mg mL^−1^ were used to prepare the sensors. It was found the amperommetric responses of the AgNPs-MWCNT/GCE increased with the increase of the amount of AgNPs-MWCNT on the GCE surface, a further concentration increase resulted in signal saturation. As a result, 4 mg mL^−1^ of AgNPs-MWCNT was used to prepare the sensor. Figure 4b shows the typical current-time (i–t) curve of AgNPs-MWCNT/GCE with the successive addition of H_2_O_2_ in 0.1 M PBS (pH 7.4) at room temperature under nitrogen atmosphere. One can see that the responsive current of AgNPs-MWCNT/GCE increases rapidly with the successive injection of H_2_O_2_ and reaches steady state within 3s. The sensitive and fast response of H_2_O_2_ on the AgNPs-MWCNT/GCE may be due to the large surface area of the AgNPs-MWCNT nanocomposite, which possesses a large amount of electrocatalytic active sites to improve the sensing sensitivity [33]. Figure 4c shows the relationship between the concentration of H_2_O_2_ and the step current. There are two linear ranges from 1 to 10 μM (*I_r_*(μA) = −0.1806 C (μM) − 5.4262, *R^2^* = 0.9937), and from 10 to 1000 μM (*I_r_*(μA) = −0.0125 C (μM) − 7.6935, *R^2^* = 0.9975). According to the linear regression equation, the detection limit of H_2_O_2_ on AgNPs-MWCNT/GCE is estimated to be 0.38 μM (S/N = 3). Moreover, the sensitivity of the detection was about 2556 μA cm^−2^ mM^−1^ at the low concentration range (1 to 10 μM). The above results indicate that the AgNPs-MWCNT nanocomposite possesses excellent electrocatalytic property to H_2_O_2_, which can be ascribed to the following reasons—first, both AgNPs and MWCNTs have excellent electron transfer efficiency, the hybrid nanocomposite (AgNPs-MWCNT) has higher electron transfer efficiency, which can enhance the reaction rate of hydrogen peroxide; second, the synthesized AgNPs possess a smaller size, well dispersion, and good uniformity deposited on MWCNT surface, which can enlarge the surface area and increase the electrocatalytic active sites of the nanocomposite; third, the synergistic effect was generated between AgNPs and CNTs, which can improve the sensitivity of the H_2_O_2_ detection [34]. Table 1 displays the detection limits and linear ranges of AgNPs–MWCNT/GCE and the other related sensors in literature [35,36,37]. The limit of detection (LOD) of the AgNPs–MWCNT sensor in this work is comparable or better than that of the other nonenzymatic H_2_O_2_ sensors.

### 2.4. Selectivity, Reproducibility, and Stability of the AgNPs-MWCNT/GCE Sensor

**Figure 5:**
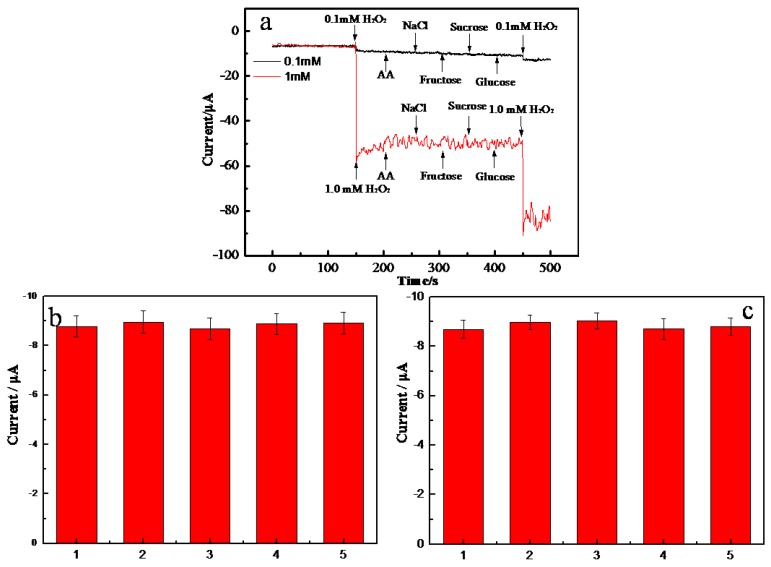
(**a**) Chronoamperometric curve (black) of the AgNPs-MWCNT/GCE in response to the successive addition of 0.1 mM H_2_O_2_, 5 mM ascorbic acid, 5 mM NaCl, 5 mM fructose, 5 mM sucrose, 5 mM glucose and 0.1 mM H_2_O_2_ in 0.1 M PBS at a working potential of −0.5 V; chronoamperometric curve (red) of the AgNPs-MWCNT/GCE in response to the successive addition of 1 mM H_2_O_2_, 5 mM ascorbic acid, 5 mM NaCl, 5 mM fructose, 5 mM sucrose, 5 mM glucose and 1 mM H_2_O_2_ in PBS at a working potential of −0.5 V. (**b**) Current responses of five equally fabricated sensors to 0.1mM H_2_O_2_. (**c**) Current responses of the AgNPs-MWCNT/GCE biosensor to 0.5 mM H_2_O_2_. Amperommetric measurements were performed in 5 days using the same sensor.

Figure 5a shows the amperommetric responses of 0.1 mM and 1.0 mM H_2_O_2_ in the absence and presence of 0.5 M of potential interferences including ascorbic acid, NaCl, fructose, sucrose, and glucose (last injection). One can see that similar responsive currents were obtained in the absence and presence of interferences. In addition, there was no responsive current observed when injecting the interferences in the absence of H_2_O_2_. The above results indicate that the AgNPs-MWCNT/GCE sensor has excellent selectivity toward H_2_O_2_ detection. The reproducibility of the AgNPs-MWCNT/GCE sensor was studied by measuring 0.1 mM H_2_O_2_ with five electrodes fabricated at the same batch (Figure 5b). Similar responsive currents were obtained and the relative standard deviation (RSD) of the five measurements was 1.2%, indicating the excellent reproducibility of the sensor. The stability of AgNPs-MWCNT/GCE was tested by measuring the responsive current of 0.1 mM H_2_O_2_ for 5 days, and the sensor was kept in room temperature. As shown in Figure 5c, no obvious current difference was observed in five days, and the RSD of the measured currents was 1.8%. The above experimental results indicate that the AgNPs-MWCNT/GCE has good stability.

### 2.5. Detection of H_2_O_2_ in Human Blood Serum

The AgNPs-MWCNT/GCE sensor was applied to detect H_2_O_2_ in human blood serum (HBS). The HBS was diluted 50 times by 0.1 M PBS (pH 7.4), and the H_2_O_2_ standards were added to the diluted HBS. The concentrations of H_2_O_2_ in the diluted HBS were fixed at 5.0, 50, and 500 μM, respectively. Each sample was detected three times at same condition. The data are displayed in Table 2. The recovery rates of H_2_O_2_ in the diluted HBS were 90.2, 92.8, and 96.8% respectively; and the RSD were 1.75, 0.47, and 0.61% respectively. The results indicated that the fabricated AgNPs-MWCNT/GC sensor has good ability for H_2_O_2_ detection in practical application.

## 3. Materials and Methods

### 3.1. Materials and Instruments

MWCNT was purchased from Nanjing XFNano Materials Technology Co., Ltd. (Nanjing, China). Silver nitrate (AgNO_3_, 99.5%), sodium citrate, glucose, ascorbic acid, fructose, phosphate buffer saline (PBS, PH 7.4, 0.1 M), and lactose were purchased from Sigma (Shanghai, China). Sulfuric acid (H_2_SO_4_, 98%), nitric acid (HNO_3_, 98%), and hydrogen peroxide (H_2_O_2_) were purchased from Sinopharm Chemical Reagent Co., Ltd. (Beijing, China). Human blood plasma was purchased from Senbeijia Biotechnology Co., Ltd. (Nanjing, China). All the reagents were of analytical grade and used as received without further purification. Ultrapure water (resistance >18 MΩ cm^−1^, Milli-Q purification system) was used in all of the experiments.

Electrochemical studies were performed on a CHI 660D electrochemical system (Chenhua, Shanghai, China). The three-electrode system consists of a AgNPs-MWCNT modified glassy carbon working electrode, a Ag/AgCl/KCl (saturated) reference electrode, and a platinum wire counter electrode. The morphology and size of the CNT and AgNPs-MWCNT were characterized by TEM (JEOL, Kyoto, Japan, 2100F, 200KV). XRD measurements were performed on a Shimadzu XRD-6000 (Shimadzu, Kyoto, Japan) using Cu Kα radiation (1.5406 Å) and operated at 36 kV and 20 mA. All the experiments were carried out at room temperature. The content of Ag in the AgNPs-MWCNT nanocomposite was determined by ICP-AES (TJA IRIS Advantage ER/S, Thermo, Franklin, MA, USA).

### 3.2. Synthesis of AgNPs-MWCNT Nanocomposite

The MWCNTs were shortened and functionalized with the previous reported method [38]. Briefly, 40 mg of MWCNTs, 19.2 mL of concentrated sulfuric acid, and 6.4 mL of concentrated nitric acid were added to a glass flask with a volume of 50 mL. The mixture was sonicated for 6 h at room temperature and separated through centrifugation at 8,000 rpm for 10min; then the MWCNT pellet was washed several times with deionized water and ethanol, respectively. The final product was dried for 24 h at 35 °C under nitrogen atmosphere. 

The AgNPs-MWCNT nanocomposite was prepared by a reported method with a slight modification [39]. Three milligrams of the functionalized MWCNTs and 600 μL of 0.1 g mL^−1^ sodium citrate solution were dispersed in 30 mL of deionized water, the mixture was heated at 100 °C for 5 h under vigorous stirring. Then, the mixture was cooled to 60 °C, and the 96.6 μL of 0.01 M silver nitrate solution was added to the mixture. The mixture solution continued to react for 5 h at 60 °C under vigorous stirring. After the system was cooled to room temperature, the resulting product (AgNPs-MWCNT) was separated though centrifugation, washed three times with deionized water and ethanol, and dried for 48 h at 35 °C under nitrogen atmosphere.

### 3.3. Preparation of the AgNPs-MWCNT/GCE

The GCE (Φ = 3 mm) was polished with 0.5 and 0.05 μm alumina slurries, respectively, and followed by sonication in deionized water and ethanol. The electrode was dried under nitrogen atmosphere. A 10 μL of AgNPs-MWCNT aqueous solution (4 mg mL^−1^) was dropped on the cleaned GCE surface and dried in air, then a 10 μL of Nafion (0.05 wt%) was dropped again and dried at room temperature. The MWCNT-modified GCE was prepared in the same way.

### 3.4. Electrochemical Measurements

A three-electrode cell contains AgNPs-MWCNT/GCE as working electrode, platinum wire as the counter electrode, and Ag/AgCl as the reference electrode. Cyclic voltammograms (CV) were obtained at the potential range between 0.2 and −1.6 V in 0.1 M PBS at pH 7.4 at a scan rate of 100 mV s^−1^. Amperommetric experiments were carried out in 20.0 mL of 0.1 M PBS (pH 7.4) with the successive addition of H_2_O_2_ under mild stirring. The electrode potential was set at −0.5 V (vs. Ag/AgCl). Before the experiment, all the PBS solution was purged with high-purity nitrogen for at least 30 min.

## 4. Conclusions

The AgNPs-MWCNT nanocomposite were successfully prepared through a simple and facile two-step method. The advantage of this method was that the silver nanoparticles were highly dispersed on the functionalized carbon nanotubes. The AgNPs-MWCNT nanocomposite was used to prepare a nonenzymatic electrochemical sensor for the detection of H_2_O_2_ with wide linear range, low detection limit, and excellent reproducibility and stability. The sensor was applied to detect H_2_O_2_ in spiked human blood serum with satisfactory results.

## Figures and Tables

**Table 1:** Performance comparisons of the H_2_O_2_ sensors fabricated with different materials.

Modify Electrode	Linear Range (μM)	LOD (μM)	Ref.
PtNPs-MWCNTs	2–3800	0.7	[35]
AuNPs-MWCNT	20–300	0.4	[36]
AgNCs-GO	20–10000	3.0	[13]
Ag NPs-MWCNT	50–17000	0.5	[37]
AgNPs-MWCNT-rGO	100–100000	0.9	[24]
AgNPs-MWCNT	1–1000	0.38	This work

**Table 2:** Results of standard addition and recovery in human blood serum.

Samples	Added (μM)	Founded (μM)	Recovery (%)	RSD (%)
1	5.0	4.5	90.2	1.75
2	100	92.8	92.8	0.47
3	500	483.8	96.8	0.61
